# Neuroprotective effects of microRNA‐211‐5p on chronic stress‐induced neuronal apoptosis and depression‐like behaviours

**DOI:** 10.1111/jcmm.16716

**Published:** 2021-06-13

**Authors:** Jie Shen, Ping Zhang, Ye Li, Cuiqin Fan, Tian Lan, Wenjing Wang, Shu Yan Yu

**Affiliations:** ^1^ Department of Neurosurgery Qilu Hospital of Shandong University Jinan China; ^2^ Department of Physiology School of Basic Medical Sciences Cheeloo College of Medicine Shandong University Jinan China; ^3^ Shandong Key Laboratory of Mental Disorders School of Basic Medical Sciences Cheeloo College of Medicine Shandong University Jinan China

**Keywords:** apoptosis, CUMS, depression, hippocampus, microRNA‐211‐5p

## Abstract

Findings from recent studies have revealed that microRNAs (miRNAs) are related to numerous neurological disorders. However, whether miRNAs regulate neuronal anomalies involved in the pathogenesis of depression remain unclear. In the present study, we screened miRNA expression profiles in the CA1 hippocampus of a rat model of depression and found that a specific miRNA, microRNA‐211‐5p, was significantly down‐regulated in depressed rats. When miR‐211‐5p was up‐regulated in these rats, neuronal apoptosis within the CA1 area was suppressed, effects which were accompanied with an amelioration of depression‐like behaviours in these rats. These neuroprotective effects of miR‐211‐5p in depressed rats appear to result through suppression of the Dyrk1A/ASK1/JNK signalling pathway within the CA1 area. In further support of this proposal are the findings that knock‐down of miR‐211‐5p within the CA1 area of normal rats activated the Dyrk1A/ASK1/JNK pathway, resulting in the promotion of neuronal apoptosis and display of depression‐like behaviours in these rats. Taken together, these results demonstrate that deficits in miR‐211‐5p contribute to neuronal apoptosis and thus depression‐like behaviours in rats. Therefore, the miR‐211‐5p/Dyrk1A pathway may be critically involved in the pathogenesis of depression and serve as a potential therapeutic target for the treatment of depression.

## 1 INTRODUCTION

Depression is a major psychiatric disorder associated with structural and functional changes within specific brain regions.^1^, ^2^, ^3^ However, the underlying pathophysiological mechanisms involved in this disorder, and thus the potential for corresponding therapeutic strategies, are not fully understood. Previous evidence has indicated that stressful stimuli‐induced modulators can contribute to cell apoptosis, which may then be a critical neural process involved in the progression of neuronal injury and consequent depression‐like behaviours.^4^, ^5^, ^6^ Apoptosis is a complicated process which plays a central role in controlling cell numbers and tissue size to maintain a normal structural and functional homeostasis, with disruptions in this homeostasis believed to play an essential role in a variety of diseases.^7^ Recently, results from clinical studies have revealed that inflammation and its consequent effects upon neuronal loss were observed in specific depression‐associated brain regions of patients with advanced depression.^8^, ^9^, ^10^ Consistently, accumulating evidence from animal models of depression have indicated that stressful stimuli produce neuronal damage accompanied with an increase in apoptosis within the hippocampus and the display of depression‐like behaviours.^11^, ^12^ However, details regarding the molecular mechanisms of these neurological processes, in particular whether this neuronal apoptosis is involved in the pathological damage resulting in depression, are far from being fully understood. Therefore, identification of the effects and mechanisms underlying the neuronal apoptosis associated with the pathogenesis of depression may provide critical information needed to understand and develop more effective treatments for this disorder.

Recently, microRNAs (miRNAs) have been suggested to be important factors in the epigenetic control of neural development and function.^13^, ^14^ Dysfunction in specific miRNAs contributes to many neurological diseases,^15^, ^16^, ^17^ including major depression disorder (MDD).^18^ Findings from these clinical studies reveal that various miRNAs are differentially expressed in cerebrospinal fluid, serum and the depression‐associated brain regions of MDD patients, suggesting that miRNAs might be important participants in the pathogenesis of depression. However, detailed molecular pathways underlying the neural mechanisms of apoptosis in depression, in particular, in the chronic unpredictable mild stress (CUMS)–induced rat model of depression, remain to be determined. Moreover, whether targeting the disruption in miRNA systems can be used as a potential therapeutic strategy for depression treatment is also currently unknown.

Therefore, in the present study, we investigated the involvement of chronic stress–induced neuronal apoptosis in a CUMS rat model of depression and examined possible molecular mechanisms mediating the progress of apoptosis in the CA1 region of the hippocampus. With use of the RNA‐sequencing assay, we identified a differential increase in miR‐211‐5p in this CUMS‐induced rat model of depression and assessed the role of its downstream signalling pathway, Dyrk1A (dual‐specificity tyrosine phosphorylation‐regulated kinase 1A)/ASK1 (apoptosis signal‐regulating kinase 1)/JNK/p38, as related to the neuronal apoptosis and display of depression‐like behaviours in these rats. These findings have the potential to identify a novel regimen for use in the treatment of depression.

## 2 MATERIALS AND METHODS

### 2.1 Experimental animals

Forty‐two healthy male Wistar rats (5‐6 weeks old; 160‐180 g bodyweight) were provided by the Experimental Animal Center of Shandong University. The rats were housed one per cage and maintained under conditions of 22‐25℃, an indoor humidity of 35%‐40% and a 12‐h light‐dark cycle. They were permitted free access to standard feed and water, and the colony room was regularly disinfected with use of ultraviolet irradiation. The experiments conducted in this study were approved by the Ethics Committee of Shandong University and implemented in strict accordance with the care and treatment of experimental animals and related regulations of the National Institutes of Health.

### 2.2 Agents and antibodies

The miRNAs—miRNA‐211‐5p, miRNA‐1298, miRNA‐204‐5p and miRNA‐34b‐3p—were obtained from RiboBio. The monoclonal rabbit anti‐Dyrk1A (8765), anti‐phospho‐JNK (4668), anti‐phospho‐ASK1 (3764s), anti‐phospho‐p38 (9211), anti‐β‐actin (4970) and anti‐Cleaved Caspase3 (9661) were purchased from Cell Signaling. The anti‐NeuN was purchased from Abcom (ab104224). The polyclonal goat anti‐rabbit secondary antibody was purchased from Beijing Zhongshan Golden Bridge. Rhodamine (TRITC)‐conjugated goat anti‐rabbit IgG and AMCA‐conjugated Affinipure goat anti‐mouse IgG (H + L) were obtained from the Proteintech Group. Hoechst 33 258 (C0031) was obtained from Solarbio. Constructed adeno‐associated viruses HBAAV2/9‐rna‐miR‐211‐5p‐sponge‐GFP (AAV‐miR‐211‐5p sponge) (#GMDV0202715) and HBAAV2/9‐rna‐miR‐211‐5p‐GFP (AAV‐miR‐211‐ 5p) (#GMUV0202717) were obtained from GeneChemCo. In this study, viral vectors without target gene sequences were used as AAV injection controls.

### 2.3 CUMS model

The chronic unpredictable mild stress (CUMS) rat model of depression has been described previously.^19^ Briefly, rats were exposed to a series of stressor events including the following: 24 hours food deprivation followed by 24 hours water deprivation, tail clamping for 3 minutes, day and night inversion, wet cage environment (wet sawdust as litter) for 24 hours, physical restraint (2 hours), unpleasant odour, forced swimming in ice water for 3 minutes and tilted cage for 24 hours. These 8 stressors were randomly applied for a 6‐week period. Normal (control) rats were maintained as described above and were not exposed to any of these stressors over this 6‐week period.

### 2.4 Behavioural tests

Behavioural tests used to assess the display of depression included the sucrose preference test (SPT) and forced swimming test (FST). All behavioural responses were recorded by observers blind as to treatment condition of the animals.

#### 2.4.1 Sucrose preference (SPT)

The demonstration of a preference for sugar water is one method that can be used to test for the core symptoms of a lack of pleasure in depressed rats. CUMS and normal rats were tested for their sugar water preference.^20^ For the first 24‐hours period (day 1), two bottles containing 100 mL of a 1% (mass/volume) sucrose solution were placed in each cage. On day 2, one bottle containing 100 mL of sucrose and one bottle with ordinary water were simultaneously placed in the cage for 24 hours with the positions of the two bottles changed after 12 hours. On day 3, the rats were food and water deprived for 24 hours, and on day 4, the sucrose preference test was conducted. The test duration was 180 minutes and consisted of simultaneously placing one bottle containing 100 mL of sucrose and one bottle with ordinary water in each cage, with the positions changed after 90 minutes. The consumption of the sugar water and ordinary water was recorded over this 180‐minutes period. A sucrose preference was defined as the amount of sugar water intake/(sugar water intake +ordinary water intake) × 100%.

#### 2.4.2 Forced swimming test (FST)

After completion of the SPT, the forced swimming test was administered as described previously.^21^ Briefly, the experimental chamber consisted of a plastic cylinder with a height of 80 cm, diameter of 40 cm and containing a 20‐25 cm height of water (25℃), which prevented the tail of the rat from touching the bottom of the cylinder. The rats were initially placed in the chamber for a swimming training session of 15 minutes with the forced swimming behavioural test then conducted 24 hours later. For the test, the water temperature and depth remained unchanged and the total test time was 5 minutes. The immobility and swimming times of each rat were recorded during this 5 minutes test. The immobility time refers to the amount of time that the rat floats on the surface of the water, only maintaining its head above the water. Increased immobility times and decreased swimming times provide an index of ‘behavior despair’. During the test period, a silent and light‐controlled surrounding environment was maintained to avoid any external factors from interfering with the experimental results.

### 2.5 miRNA sequencing and dual‐luciferase assay

Total RNA was isolated from CA1 tissues of normal and CUMS rats using the TRIzol kit (Invitrogen). RNA integrity was accessed with use of Agilent 2200 TapeStation (Agilent Technologies). Purified library products were sequenced using the HiSeq 2500 and HiSeq 3000 (Illumina) platform by the RiboBio Biotechnology Company. Dual‐luciferase assay was used to assess the target gene of miR‐211‐5p. The luciferase reporter plasmid (pmirGLO‐Dyrk1A or pmirGLO‐Dyrk1A‐MUT recombinant vector) was co‐transfected with miR‐211‐5p mimic or miR‐NC into HEK‐293T cells using Lipofectamine 2000 (Invitrogen, 11668027) for 48 hours and assayed for firefly activity and renilla luciferase activity.

### 2.6 Brain stereotaxic injection of the AAV virus

After completion of these initial baseline behavioural tests, rats were selected for virus injection. The rats were anaesthetized using an intraperitoneal injection of chloral hydrate (4 mL/kg) and then fixed onto a brain stereotaxic instrument. The injection site of the hippocampal CA1 area was according to coordinates of the Rat Brain Atlas (from bregma: −3.24 mm; medial/lateral: ± 2.5 mm; dorsal/ventral: −3.1 mm). Three microliters of concentrated AAV virus (~10^12^ infection units per mL) was injected bilaterally into the hippocampal CA1 area over a 30‐minutes period. The SPT and FST behavioural tests were then administered at two weeks after the virus infection, and only rats receiving a successful injection of the AAV virus were selected for further experiments.

### 2.7 Extraction of brain tissue

After completion of the post‐AVV virus injection behavioural tests, rats with successful responses received an intraperitoneal injection of chloral hydrate (4 mL/kg) and while under deep anaesthesia were quickly decapitated. The brain was removed, and the hippocampal CA1 area was isolated (N = 6/group).

### 2.8 Immunofluorescence staining and confocal microscopy

To obtain frozen brain sections for immunofluorescent assay, rats with correct AVV infusion sites were injected intraperitoneally with chloral hydrate (4 mL/kg) at 24 hours after completion of behavioural tests. The heart was initially perfused with normal saline followed by a slow perfusion with 4% paraformaldehyde (PFA). The brain was removed and placed in 4% PFA at 4℃ overnight. The brains were then subjected to sucrose gradient sedimentation, embedding and sectioning on a frozen microtome to obtain brain slices for immunofluorescence assay (N = 6/group). After brain sections (30 μm) were equilibrated to room temperature for ten minutes, the slices were placed in an immunofluorescent blocking solution and incubated for 60 minutes at room temperature. The primary antibody (1:100) was added to the brain slices and incubated at 4℃ overnight. After washing 3 times with PBS buffer, the fluorescent secondary antibody (1:200) was added and incubated for 60 minutes at 37℃ in a constant temperature shaker (avoiding light). The slices were then washed 3 times with PBS buffer, 75% glycerine sealed and then viewed as soon as possible. Immunofluorescent stained images were acquired with use of a LSM780 laser scanning confocal microscope system (ZEISS). At least six representative images were obtained from each rat for analysis by Image‐Pro plus 6.0 software.

### 2.9 Western blot analysis

The hippocampus was isolated and weighed and then combined with RIPA lysate, protein phosphatase inhibitor mixture (PI) and protease inhibitor PMSF (100:1:1, Beyotime) according to a weight‐to‐volume ratio. After homogenization, samples were centrifuged at 12,000 rpm for 20 minutes in 4℃. A sample of the supernatant was removed and added to a loading buffer (Beyotime) at a ratio of 1:1. The sample was agitated, placed in a bath for 10 minutes at 100℃ then immediately cooled on ice and stored at −20℃. After electrophoretical separation on (SDS‐PAGE) electrophoresis, the samples were transferred to PVDF membranes, blocked and incubated with the primary antibody (1:1000) at 4℃ overnight. The samples were then washed three times with TBST and incubated with the secondary antibody (1:10 000) at room temperature for 1 hour, using an ECL Luminescent Solution Display Protein system (Beyotime). Proteins were analysed using ImageJ software (NIH, Scion Corporation).

### 2.10 Reverse transcription PCR and real‐time quantitative PCR

#### 2.10.1 Reverse transcription PCR (RT‐PCR)

Total RNA was extracted from the CA1 region with use of the RNA Quick Extraction Kit (AXYGEN). Primers were purchased from BGI. RNA (1 μg of total) was used as a template for reverse transcription with use of the Toyobo Kit and amplified with TransGen Mix. PCR products were analysed using electrophoresis on a 1.5% agarose gel and were assessed using the Gel Image Analysis System (Bio‐Rad).

#### 2.10.2 Real‐time quantitative PCR

Total RNA was extracted from the CA1 region with use of the RNA Quick Extraction Kit (AXYGEN). Primers were purchased from RiboBio. Reverse transcription and amplification were performed according to directions of the miDETECT A TrackTM miRNA qRT‐PCR Starter Kit from RiboBio and real‐time quantitative PCR was analysed using a Bio‐Rad iCycler system (Bio‐Rad, Hercules). Data were analysed using SPSS version 13.0 software.

### 2.11 Statistical analysis

Data were analysed using the SPSS version 14.0 software program (SPSS Inc). Comparisons were performed using one‐ or two‐way analysis of variance (ANOVA) with the LSD t test used for post hoc pairwise comparisons. Data were expressed as means ±standard deviations, and a *P* < .05 was required for results to be considered as statistically significant.

## 3 RESULTS

### 3.1 MiR‐211‐5p is down‐regulated in the hippocampal CA1 region of CUMS rats

**FIGURE 1:**
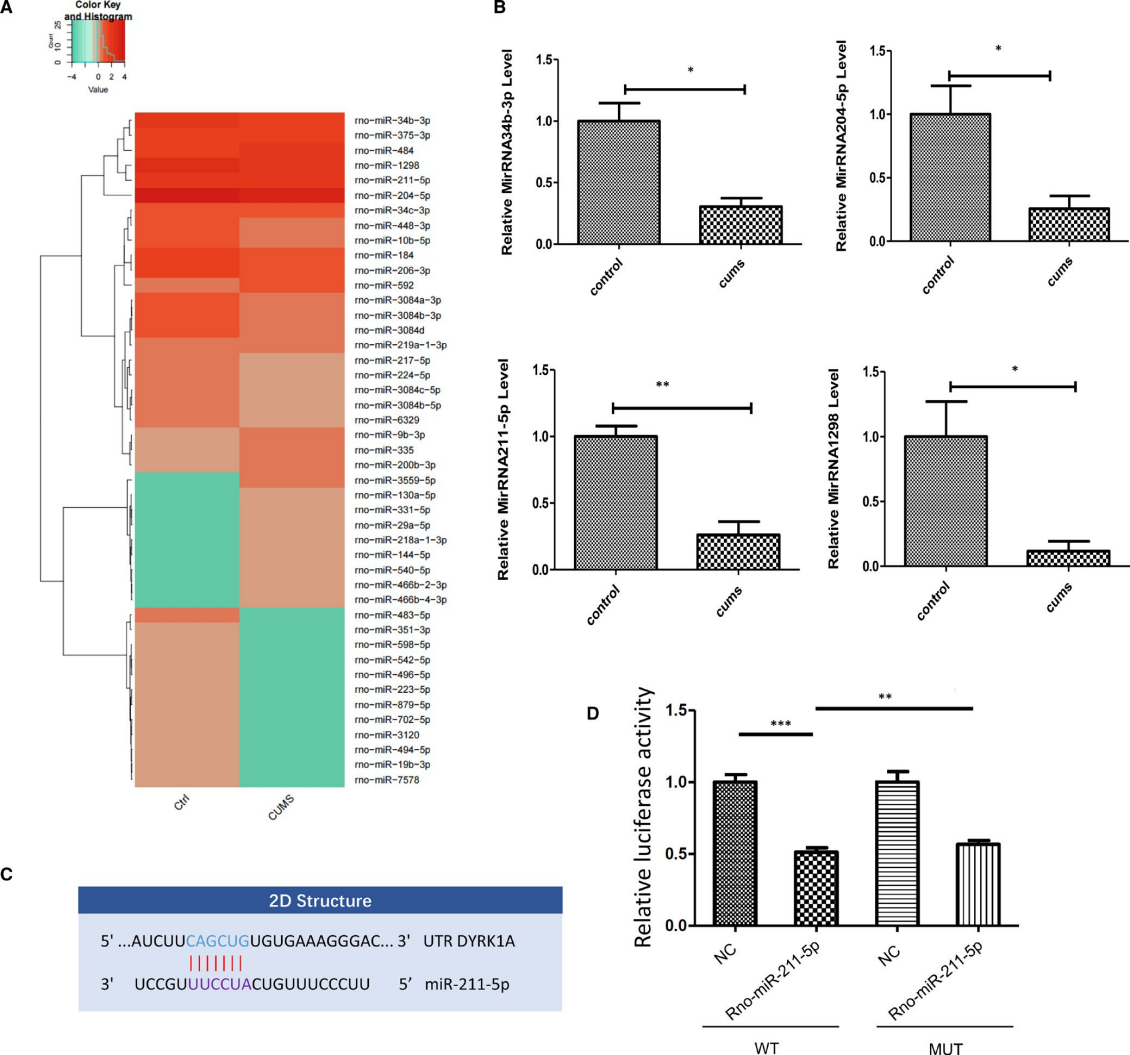
MiRNA expression profiles of CA1 tissue and validation of target genes of miR‐211‐5p. A, Heatmap diagram of differential miRNA expression levels by sequencing on the Illumina HiSeq 2500 platform. B, Quantitative real‐time PCR was used to validate expressions of miR‐211‐5p, miR‐1298, miR‐204‐5p and miR‐34b‐3p. N = 6 per group. Data are presented as the means ± SEMs. *P < .05, **P < .01. C, Putative seed‐matching sites between miR‐211‐5p and DYRK1A. (D) Luciferase reporter assay was used to detect relative luciferase activities of WT and MUT DYRK1A reporters. (**P < .01; **P < .01)

High‐throughput sequencing of small RNAs was used to identify differentially expressed miRNAs in the hippocampal CA1 area (Figure 1A), while real‐time PCR was used to verify miRNA expressions of the miRNAs, miR‐211‐5p, miR‐1298, miR‐204‐5p and miR‐34b‐3p (Figure 1B). The dual‐specificity tyrosine phosphorylation‐regulated kinase 1A (Dyrk1A) was predicted as one of the target genes of miR‐211‐5p as based on the TargetScan database (Figure 1C). Moreover, the dual‐luciferase reporter assay demonstrated that binding sites exist between Dyrk1A mRNA and miR‐211‐5p (Figure 1D).

### 3.2 Up‐regulation of miR‐211‐5p alleviates depression‐like behaviours in CUMS rats

**FIGURE 2:**
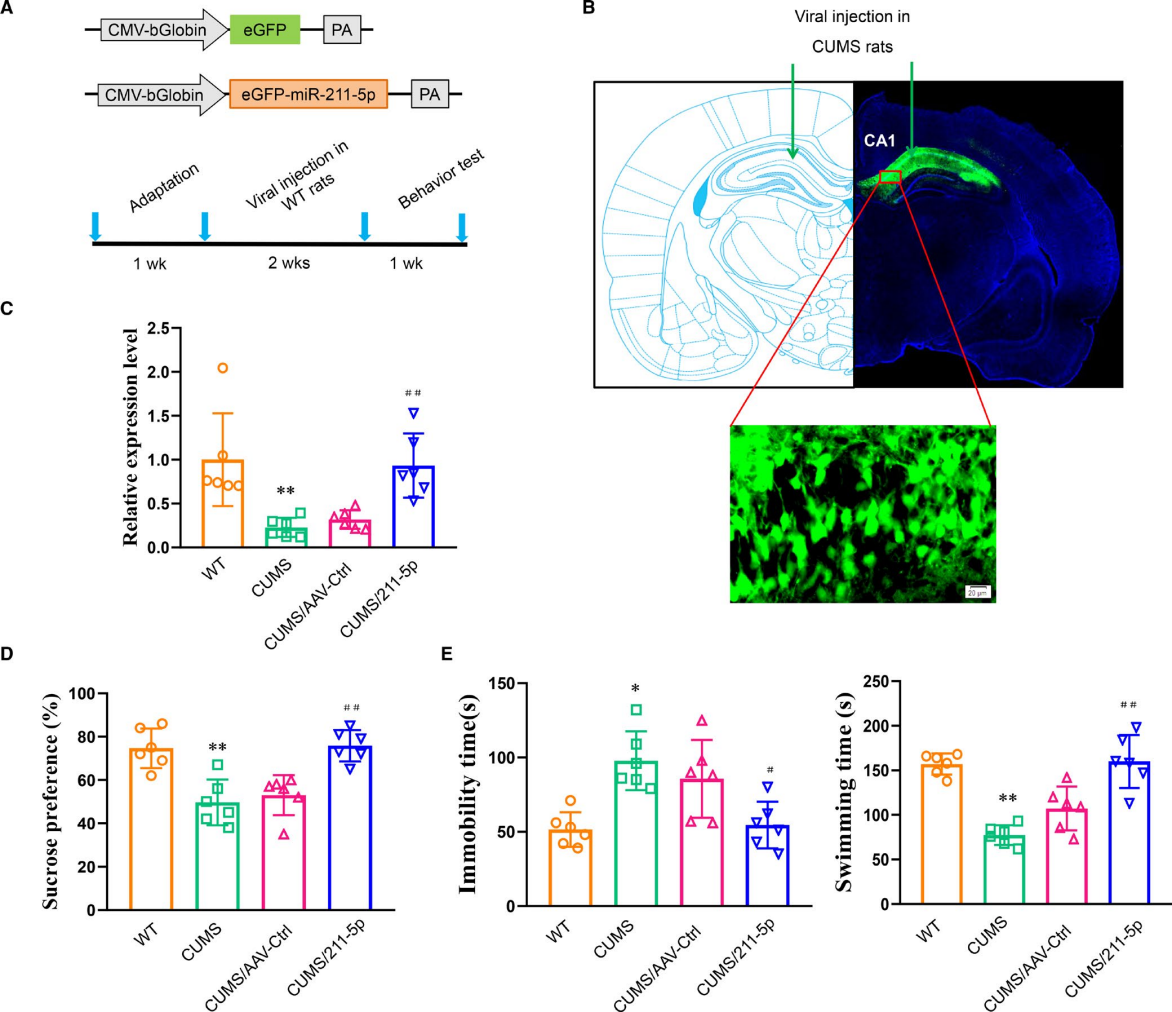
Up‐regulation of miR‐211‐5p within the CA1 region of CUMS rats ameliorates depression‐like behaviours. A, Construct of AAV‐miR‐211‐5p and experimental paradigm. B, Representative site of bilateral viral injections into the CA1 hippocampus. C, Validation of miR‐211‐5p overexpression efficiency in CA1 regions showed that the expression level of miR‐211‐5p was significantly increased in CUMS rats. Up‐regulation of miR‐211‐5p in CUMS rats (D) increased sucrose consumption and (E) decreased immobility times and increased swimming times. N = 6 per group. Data are presented as the means ± SEMs. *P < .05, **P < .01 vs. wild type; ^#^ *P* < .05, ^##^ *P* < .01 vs. AAV‐control (CUMS+AAV‐Ctrl). WT, wide type

In order to examine whether up‐regulation of miR‐211‐5p would rescue depression‐like behaviours in CUMS rats, the AAV‐rno‐miR‐211‐5p virus was constructed (Figure 2A) and bilaterally infused into the CA1 region (Figure 2B). The overexpression efficiency of miR‐211‐5p was assessed with use of qRT‐PCR and results showed that the expression level of miR‐211‐5p was significantly increased after viral injection as compared to that of the control group (*P* < .01, Figure 2C). Behavioural tests were conducted at 14 days after AAV‐miR‐211‐5p virus infusion. An overall statistically significant difference in the per cent of sucrose consumption was obtained among the four groups. Post hoc analysis revealed that CUMS rats showed a decrease in consumption per cent as compared to the non‐stressed control group (*P* < .05). This decrease in CUMS rats was effectively reversed by an up‐regulation of miR‐211‐5p (*P* < .05, Figure 2D). Moreover, in the forced swim test, an up‐regulation of miR‐211‐5p significantly decreased the immobility times and increased swimming times in these CUMS rats (*P* < .05, Figure 2E).

### 3.3 Up‐regulation of miR‐211‐5p suppresses neuronal apoptosis in CUMS rats

**FIGURE 3:**
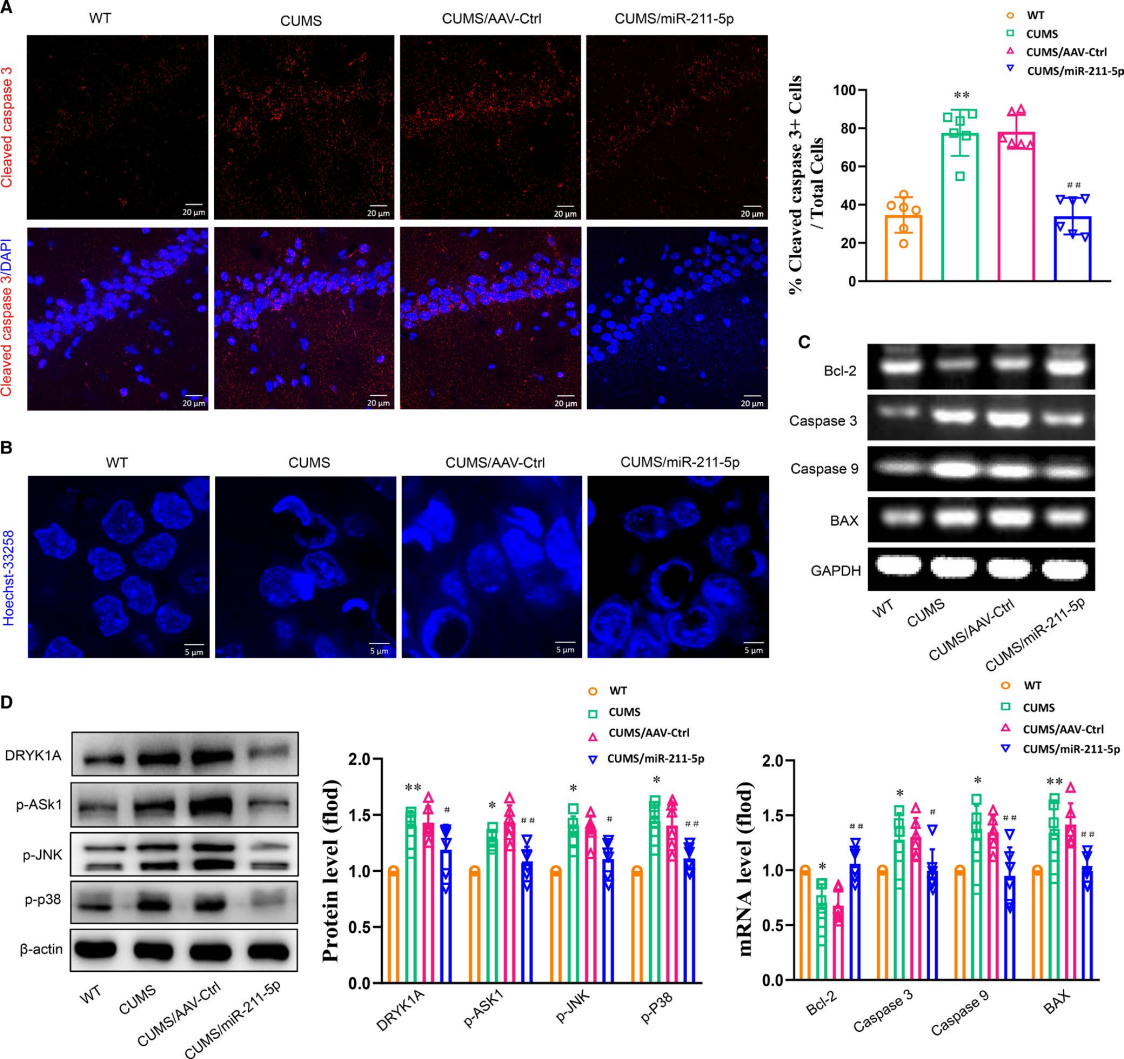
Up‐regulation of miR‐211‐5p decreased neuronal apoptosis in CUMS rats. A, Representative confocal microscopic images showing positive cells labelled with cleaved caspase 3 within the CA1 region. Scale bar is 20 µm. B, Representative images of Hoechst‐33258 staining to observe morphological changes in nuclei. Scale bar is 5 µm. C, mRNA expression levels of Bcl2, Bax, cleaved caspase 3 and caspase 9 within the CA1 region. (D) Up‐regulation of miR‐211‐5p decreased Dyrk1A expression and phosphorylated levels of ASK1, JNK and p38 within the CA1 region. N = 6 per group. Data are presented as the means ± SEMs. *P < .05, **P < .01 vs. wild type; ^#^ *P* < .05, ^##^ *P* < .01 vs. AAV‐control (CUMS+AAV‐Ctrl). WT, wide type

Densities of positive cleaved caspase 3–labelled cells were significantly increased within the CA1 region of CUMS rats (*P* < .01, Figure 3A). In addition, nuclei of cells in the CA1 region of CUMS rats exhibited typical apoptotic changes including chromatin margination, aggregation and condensation (Figure 3B), and mRNA expression levels of pro‐apoptotic factors were significantly increased within this CA1 site (*P* < .01, Figure 3C). These changes associated with apoptosis were all reversed in response to an up‐regulation of miR‐211‐5p within the CA1 area of CUMS rats (*P* < .01). To further investigate the potential molecular mechanisms underlying these effects of miR‐211‐5p, we first assessed protein expression levels of Dyrk1A. We found that an up‐regulation of miR‐211‐5p significantly reduced the levels of Dyrk1A within the CA1 region of CUMS rats (*P* < .01; Figure 3D). Moreover, phosphorylated substrates of ASK1, JNK and p38 were all significantly decreased after up‐regulation of miR‐211‐5p in these CUMS rats (*P* < .01, Figure 3D).

### 3.4 Knock‐down of miR‐211‐5p within the CA1 region of normal rats induces depression‐like behaviours

**FIGURE 4:**
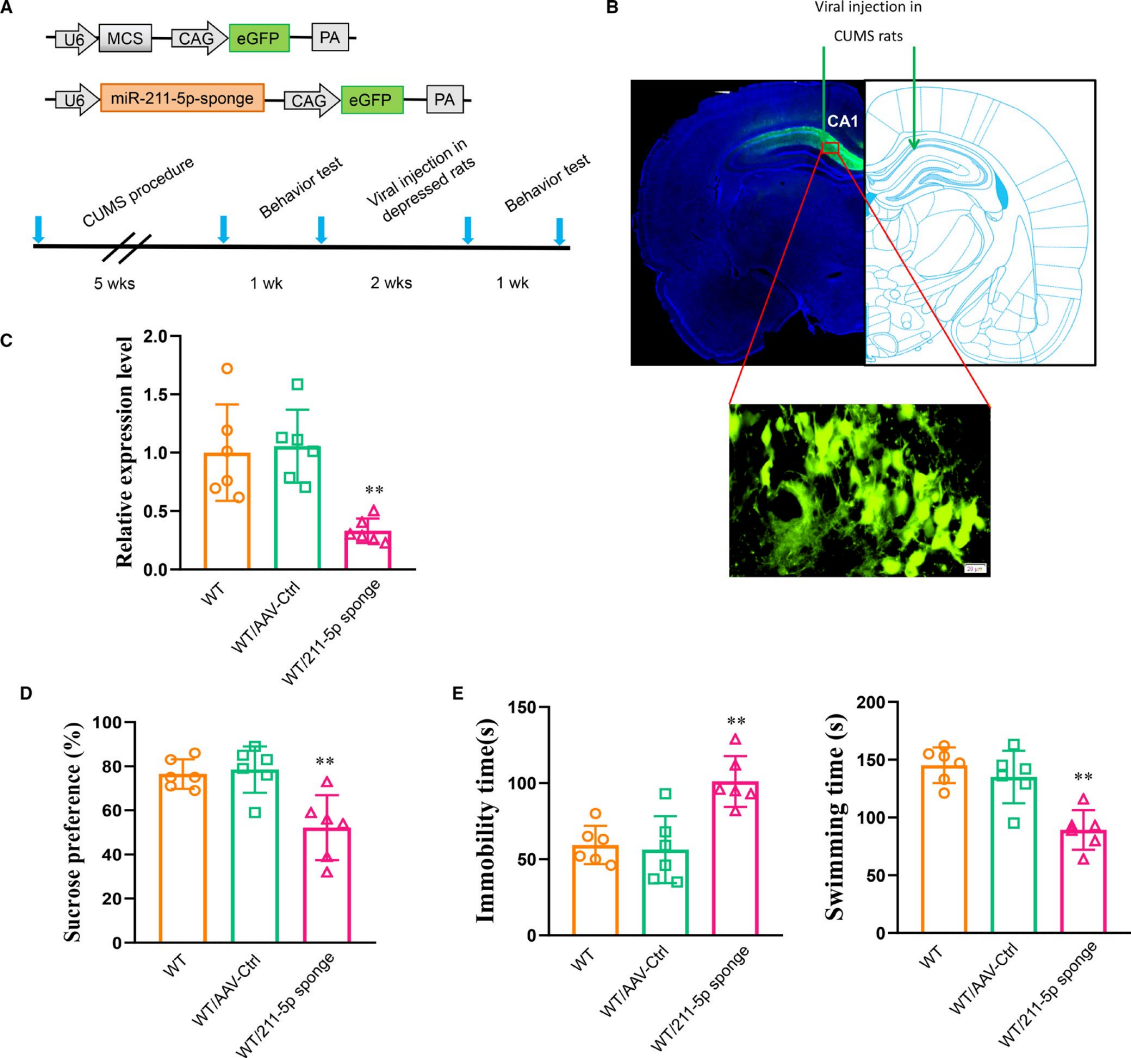
Knock‐down of miR‐211‐5p within the CA1 region of normal rats induced depression‐like behaviours. A, Schematics of AAV‐ miR‐211‐5p sponge and experimental paradigm. B, Illustration of viral injection site. C, Efficiency of miR‐211‐5p knock‐down within the CA1 region. Knock‐down of miR‐211‐5p in normal rats (D) decreased sucrose consumption and (E) increased immobility times and decreased swimming times. N = 6 per group. Data are presented as the means ± SEMs. **P < .01 vs. WT/AAV‐control (WT+AAV‐control). WT, wide type; Ctrl, control

The AAV‐miR‐211‐5p‐sponge virus was constructed and infused bilaterally into the CA1 area of normal rats (Figure 4A,B). The knock‐down efficiency was examined two weeks later, and a significant reduction was observed in miR‐211‐5p (*P* < .01, Figure 4C). This knock‐down of miR‐211‐5p within the CA1 region significantly decreased sucrose consumption (*P* < .01, Figure 4D) and increased immobility times (*P* < .01, Figure 4D) in these normal rats.

### 3.5 Knock‐down of miR‐211‐5p within the CA1 region of normal rats increases neuronal apoptosis

**FIGURE 5:**
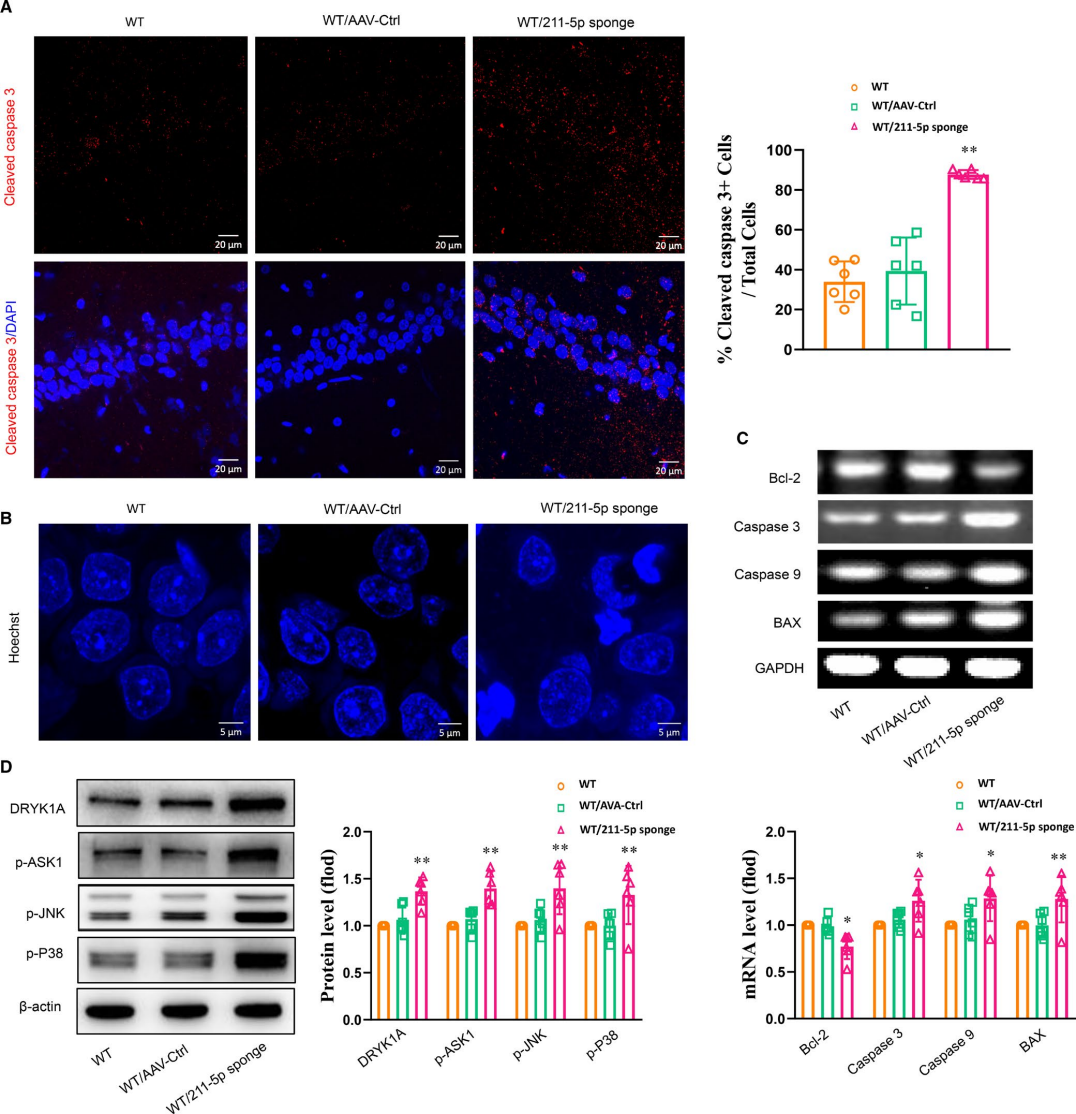
Knock‐down of miR‐211‐5p within the CA1 region in normal rats increased neuronal apoptosis. A, Representative images of cleaved caspase 3–positive cells within the CA1 region. Scale bar is 20 µm. B, Representative images of Hoechst‐33258 staining in nuclei. Scale bar is 5 µm. C, mRNA levels of apoptotic‐related factors within the CA1 region. D, Knock‐down of miR‐211‐5p increased Dyrk1A expression and phosphorylated levels of ASK1, JNK and p38 within the CA1 region. N = 6 per group. Data are presented as the means ± SEMs. *P < .05, **P < .01 vs. WT/AAV‐control (WT+AAV‐control). WT, wide type; Ctrl, control

Results from our immunofluorescent assay showed that miR‐211‐5p down‐regulation significantly increased the number of cleaved caspase 3–labelled positive cells within the CA1 region of normal rats (*P* < .01, Figure 5A). Meanwhile, Hoechst‐33258 staining revealed that knock‐down of miR‐211‐5p induced nuclear chromatin aggregation and condensation in CA1 neurons (Figure 5B), and mRNA levels of the pro‐apoptotic factors were all significantly increased by this knock‐down of miR‐211‐5p in normal rats (*P* < .01, Figure 5C). Results from Western blots showed that knock‐down of miR‐211‐5p within the CA1 area significantly increased Dyrk1A protein levels (*P* < .01, Figure 5D). Accordingly, phosphorylation levels of ASK1, JNK and p38 were all significantly increased (*P* < .05; Figure 5D). Taken together, these results suggest that the Dyrk1A /ASK1/ JNK/p38 signalling pathway may, in part, contribute to the mechanisms underlying the antidepressant‐like effects of miR‐211‐5p.

## 4 DISCUSSION

In the present study, we showed that miR‐211‐5p was significantly down‐regulated in the CA1 area within a CUMS rat model of depression and evaluated some of the downstream regulators which considered associate with neuronal apoptosis,^22^, ^23^ an important pathological process in the induction of depression‐like behaviours in rats. In specific, we found that miR‐211‐5p deficits mediated CUMS‐induced neuronal apoptosis and behavioural disorders via activation of the Dyrk1A/ASK1/JNK/P38 pathway within the CA1 area of CUMS‐exposed rats. When miR‐211‐5p was up‐regulated within the CA1 area of CUMS rats, there was a significant suppression of this apoptosis and an amelioration in the depression‐like phenotypes of these rats. Taken together, these findings reveal a neuroprotective effect of miR‐211‐5p on suppressing apoptosis during the pathogenesis of depression and may thus act as a new potential therapeutic regimen in the treatment of depression.

Results from previous clinical studies have shown that a progressive atrophy and reduction in hippocampal volume is present in patients with MDD.^24^, ^25^ Similarly, neuronal apoptosis in the CA1 area was observed in our CUMS rat model of depression as indicated by significant increases in the number of cleaved caspase 3–positive apoptotic cells. The apoptotic cells within this area of CUMS rats showed obvious nuclear damage involving chromatic agglutination, margination and nuclear karyopyknosis. Such morphological changes suggest that the neuronal apoptosis, as localized to this specific brain region, may contribute to the pathogenesis of depression. More importantly, we demonstrate that an up‐regulation of miR‐211‐5p significantly ameliorated this neuronal apoptosis within the CA1 area, effects which were accompanied with an amelioration of depression‐like behavioural disorders in these CUMS rats. To further elucidate the downstream molecular pathway of miR‐211‐5p in this depression‐related neuronal apoptosis, we used luciferase reporter assays to demonstrate that miR‐211‐5p contains conserved seed matches to Dyrk1A mRNA and targets the inhibitory translational process of Dyrk1A. Dyrk1 is a serine threonine kinase that has been suggested to be at the crossroads of many important processes in the central nervous system during development and ageing.^26^, ^27^ Abnormal expression and activity of Dyrk1A levels was found to occur in neurodegenerative diseases such as Alzheimer's and Parkinson's diseases and induces loss of neurons.^28^, ^29^ Meanwhile, the overexpression of DYRK1A in the brain damage after ischaemic stroke could initiate activity of apoptotic factors or stimulates the pro‐apoptotic pathways ASK1/JNK under oxidative stress.^30^, ^31^ As a result of these findings, selective Dyrk1A inhibitors have emerged as an attractive drug target for a variety of diseases.^32^ However, whether Dyrk1A is involved in the pathogenesis of depression, in particularly its potential role during cell death and signalling pathways, is not clearly understood. In the present study, with use of a bilaterally intracerebral injection of AAV‐miR‐211‐5p virus into the CA1 area of CUMS rats to overexpress miR‐211‐5p, we found that a significant reduction in Dyrk1A expression levels was observed, effects which paralleled the decreases in pro‐apoptotic proteins BAX, Caspase‐3 and Caspase‐9 levels along with an up‐regulation in the levels of the anti‐apoptotic protein, Bcl‐2. In addition, the increases in neuronal apoptosis within the CA1 area and depression‐like behaviours induced by CUMS were also prevented by an up‐regulation of miR‐211‐5p, suggesting that the CUMS‐induced miR‐211‐5p deficits may result in depression via elevating Dyrk1A levels. Conversely, the knock‐down of miR‐211‐5p via infusion of AAV‐miR‐211‐5p sponge virus into the CA1 region of normal rats significantly up‐regulated the expression levels of Dyrk1A and produced apoptotic phenotypes similar to that induced by CUMS exposure. Furthermore, this knock‐down of miR‐211‐5p produced effects that paralleled the CUMS‐induced overexpression of Dyrk1A and the phosphorylation levels of ASK1, JNK and p38 were obviously enhanced within the CA1 area, effects which could be alleviated by an up‐regulation of miR‐211‐5p. It has been reported that Dyrk1A directly interacts with apoptosis signal‐regulating kinase 1 (ASK1) and thus positively regulates ASK1‐mediated JNK1‐signalling under conditions of apoptotic cell death.^33^ ASK1 is a number of mitogen‐activated protein kinase (MAPK) kinases family. Accumulating evidence indicates that ASK1 activation could lead to cell death with the pathogenesis of several neurodegenerative diseases.^34^, ^35^ Therefore, in CUMS‐exposed rats, this activation of the Dyrk1A/ASK1/JNK/p38 signalling pathway may indicate one of the potential mechanisms through which the activation of neuronal apoptosis within the CA1 region eventually results in depression.

In addition to enhanced activity of the Dyrk1A/ASK1 pathway, we also found a significant increase in phosphorylation levels of the mitogen‐activated protein kinase (MAPK) pathway proteins, N‐terminal kinase (JNK) and p38 in these CUMS rats. Previous study showed that increased oxidative stress promotes activation of the JNK protein, an effector molecule of the apoptotic cascade that subsequently induces apoptotic cell death.^36^ It has been reported that p38 MAPK plays a major regulatory role in the crosstalk between the caspase‐dependent pathway and apoptosis.^37^, ^38^ Here, we demonstrate that CUMS significantly down‐regulated miR‐211‐5p, as well as increased the expression of Dyrk1A and phosphorylation levels of p38 MAPK within the CA1 area. It is considered that the p38 MAPK signalling pathway transduces signals from cell membranes to the nucleus and, in this way, participates in the cell cycle, apoptosis and proliferation.^39^ For example, p38 MAPK is crucial for caspase 3 activation and thus induces neuronal cell apoptosis as shown in the cerebral ischaemia reperfusion injury model.^40^ Therefore, together with the results found in the present study, we suggested that miR‐211‐5p deficits may up‐regulate p38 phosphorylation and thus trigger neuronal apoptosis to promote the depression‐like behaviours observed in this CUMS‐induced rat model of depression.

Altogether, our findings suggest that miR‐211‐5p/Dyrk1A pathway dysregulation may represent an important risk factor associated with the neurobiological and behavioural responses involved in depression. According to this postulate, the antidepressant‐like mechanisms of miR‐211‐5p overexpression may be due to its neuroprotective effects against neuronal apoptosis within specific sites of the brain related to depression. However, detailed molecular mechanisms regarding the pathway through which CUMS exposure may regulate miR‐211‐5p expression will require further investigation.

In conclusion, the results of this study suggest that miR‐211‐5p acts as an effective factor in ameliorating depression‐like behaviours. This capacity of miR‐211‐5p may, in part, involve distinct neuroprotective effects which have the ability to suppress neuronal apoptosis within the CA1 region as revealed in this CUMS‐induced rat model of depression. Moreover, our results reveal a possible mechanism for this antidepressant effect of miR‐211‐5p as it appears to involve the Dyrk1A/ASK1/JNK/p38 pathway as a critical downstream molecular pathway for miR‐211‐5p in mediating neuronal apoptosis. These findings provide a foundation for the development of a novel potential therapeutic regimen in the treatment of depression.

## CONFLICT OF INTEREST

The authors declare that the research was conducted in the absence of any commercial or financial relationships that could be construed as a potential conflict of interest.

## AUTHOR CONTRIBUTION

**Jie Shen:** Conceptualization (equal); Formal analysis (equal); Funding acquisition (lead); Investigation (equal); Methodology (equal); Project administration (lead); Supervision (equal); Writing‐original draft (equal); Writing‐review & editing (equal). **Ping Zhang:** Conceptualization (equal); Data curation (equal); Formal analysis (equal); Project administration (equal); Software (equal); Supervision (equal); Writing‐original draft (equal). **Ye Li:** Data curation (equal); Formal analysis (equal); Investigation (equal); Methodology (equal); Resources (equal); Software (equal); Writing‐original draft (equal). **Cuiqin Fan:** Data curation (equal); Investigation (equal); Methodology (equal). **Tian Lan:** Investigation (equal); Methodology (equal). **Wenjing Wang:** Investigation (equal). **Shuyan Yu:** Conceptualization (lead); Data curation (lead); Formal analysis (lead); Funding acquisition (lead); Investigation (lead); Methodology (lead); Project administration (lead); Resources (lead); Software (lead); Supervision (lead); Validation (lead); Visualization (lead); Writing‐original draft (lead); Writing‐review & editing (lead).

## Data Availability

The data used to support the findings of this study are available from the corresponding authors upon request.

## References

[1] Gilabert‐Juan J , Castillo‐Gomez E , Perez‐Rando M , Molto MD , Nacher J . Chronic stress induces changes in the structure of interneurons and in the expression of molecules related to neuronal structural plasticity and inhibitory neurotransmission in the amygdala of adult mice. Exp Neurol. 2011;232(1):33‐40. 21819983 10.1016/j.expneurol.2011.07.009

[2] Oh DH , Son H , Hwang S , Kim SH . Neuropathological abnormalities of astrocytes, GABAergic neurons, and pyramidal neurons in the dorsolateral prefrontal cortices of patients with major depressive disorder. Eur Neuropsychopharmacol. 2012;22(5):330‐338. 21962915 10.1016/j.euroneuro.2011.09.001

[3] Goldwater DS , Pavlides C , Hunter RG , et al. Structural and functional alterations to rat medial prefrontal cortex following chronic restraint stress and recovery. Neuroscience. 2009;164(2):798‐808. 19723561 10.1016/j.neuroscience.2009.08.053 PMC2762025

[4] Lopez‐Lopez AL , Jaime HB , Escobar Villanueva MDC , Padilla MB , Palacios GV , Aguilar FJA . Chronic unpredictable mild stress generates oxidative stress and systemic inflammation in rats. Physiol Behav. 2016;161:15‐23. 27063246 10.1016/j.physbeh.2016.03.017

[5] Fernandes J , Gupta GL . N‐acetylcysteine attenuates neuroinflammation associated depressive behavior induced by chronic unpredictable mild stress in rat. Behav Brain Res. 2019;364:356‐365. 30772427 10.1016/j.bbr.2019.02.025

[6] Maiuri MC , Zalckvar E , Kimchi A , Kroemer G . Self‐eating and self‐killing: crosstalk between autophagy and apoptosis. Nat Rev Mol Cell Biol. 2007;8(9):741‐752. 17717517 10.1038/nrm2239

[7] Hengartner MO . The biochemistry of apoptosis. Nature. 2000;407(6805):770‐776. 11048727 10.1038/35037710

[8] Cole J , Costafreda SG , McGuffin P , Fu CHY . Hippocampal atrophy in first episode depression: A meta‐analysis of magnetic resonance imaging studies. J Affect Disord. 2011;134(1–3):483‐487. 21745692 10.1016/j.jad.2011.05.057

[9] Park SC . Neurogenesis and antidepressant action. Cell Tissue Res. 2019;377(1):95‐106. 31165247 10.1007/s00441-019-03043-5

[10] Savitz J , Drevets WC . Bipolar and major depressive disorder: neuroimaging the developmental‐degenerative divide. Neurosci Biobehav Rev. 2009;33(5):699‐771. 19428491 10.1016/j.neubiorev.2009.01.004 PMC2858318

[11] Kubera M , Obuchowicz E , Goehler L , Brzeszcz J , Maes M . In animal models, psychosocial stress‐induced (neuro)inflammation, apoptosis and reduced neurogenesis are associated to the onset of depression. Prog Neuropsychopharmacol Biol Psychiatry. 2011;35(3):744‐759. 20828592 10.1016/j.pnpbp.2010.08.026

[12] Tian RH , Bai Y , Li JY , Guo KM . Reducing PRLR expression and JAK2 activity results in an increase in BDNF expression and inhibits the apoptosis of CA3 hippocampal neurons in a chronic mild stress model of depression. Brain Res. 2019;1725:146472. 31545956 10.1016/j.brainres.2019.146472

[13] Yao B , Christian KM , He C , Jin P , Ming GL , Song H . Epigenetic mechanisms in neurogenesis. Nat Rev Neurosci. 2016;17(9):537‐549. 27334043 10.1038/nrn.2016.70 PMC5610421

[14] Bredy TW , Lin Q , Wei W , Baker‐Andresen D , Mattick JS . MicroRNA regulation of neural plasticity and memory. Neurobiol Learn Mem. 2011;96(1):89‐94. 21524708 10.1016/j.nlm.2011.04.004

[15] Cao D‐D , Li LU , Chan W‐Y . MicroRNAs: key regulators in the central nervous system and their implication in neurological diseases. Int J Mol Sci. 2016;17(6):842. 10.3390/ijms17060842 PMC4926376 27240359

[16] Jovicic A , Roshan R , Moisoi N , et al. Comprehensive expression analyses of neural cell‐type‐specific miRNAs identify new determinants of the specification and maintenance of neuronal phenotypes. J Neurosci. 2013;33(12):5127‐5137. 23516279 10.1523/JNEUROSCI.0600-12.2013 PMC6705001

[17] Kawashima H , Numakawa T , Kumamaru E , et al. Glucocorticoid attenuates brain‐derived neurotrophic factor‐dependent upregulation of glutamate receptors via the suppression of microrna‐132 expression. Neuroscience. 2010;165(4):1301‐1311. 19958814 10.1016/j.neuroscience.2009.11.057

[18] Lopez JP , Fiori LM , Cruceanu C , et al. MicroRNAs 146a/b‐5 and 425‐3p and 24‐3p are markers of antidepressant response and regulate MAPK/Wnt‐system genes. Nat Commun. 2017;8:15497. 10.1038/ncomms15497 28530238 PMC5477510

[19] Willner P . Animal models as simulations of depression. Trends Pharmacol Sci. 1991;12(4):131‐136. 2063478 10.1016/0165-6147(91)90529-2

[20] Willner P . Chronic mild stress (CMS) revisited: consistency and behavioural‐neurobiological concordance in the effects of CMS. Neuropsychobiology. 2005;52(2):90‐110. 16037678 10.1159/000087097

[21] Detke MJ , Rickels M , Lucki I . Active behaviors in the rat forced swimming test differentially produced by serotonergic and noradrenergic antidepressants. Psychopharmacology. 1995;121(1):66‐72. 8539342 10.1007/BF02245592

[22] Zhang XS , Lu Y , Li W , et al. Astaxanthin ameliorates oxidative stress and neuronal apoptosis via SIRT1/NRF2/Prx2/ASK1/p38 after traumatic brain injury in mice. Br J Pharmacol. 2021;178(5):1114‐1132. 33326114 10.1111/bph.15346

[23] Wu YQ , Yuan Y , Wu CB , et al. The reciprocal causation of the ASK1‐JNK1/2 pathway and endoplasmic reticulum stress in diabetes‐induced cognitive decline. Front Cell Dev Biol. 2020;8:602. 10.3389/fcell.2020.00602 32766246 PMC7379134

[24] Koolschijn PC , van Haren NE , Lensvelt‐Mulders GJ , Hulshoff Pol HE , Kahn RS . Brain volume abnormalities in major depressive disorder: a meta‐analysis of magnetic resonance imaging studies. Hum Brain Mapp. 2009;30(11):3719‐3735. 19441021 10.1002/hbm.20801 PMC6871089

[25] Savitz J , Drevets WC , Smith CM , et al. Putative neuroprotective and neurotoxic kynurenine pathway metabolites are associated with hippocampal and amygdalar volumes in subjects with major depressive disorder. Neuropsychopharmacology. 2015;40(2):463‐471. 25074636 10.1038/npp.2014.194 PMC4443961

[26] Arbones ML , Thomazeau A , Nakano‐Kobayashi A , Hagiwara M , Delabar JM . DYRK1A and cognition: a lifelong relationship. Pharmacol Ther. 2019;194:199‐221. 30268771 10.1016/j.pharmthera.2018.09.010

[27] Guedj F , Pereira PL , Najas S , et al. DYRK1A: a master regulatory protein controlling brain growth. Neurobiol Dis. 2012;46(1):190‐203. 22293606 10.1016/j.nbd.2012.01.007

[28] Martinez‐Cue C , Rueda N . Signalling pathways implicated in Alzheimer′s Disease neurodegeneration in individuals with and without down syndrome. Int J Mol Sci. 2020;21(18):6906. 10.3390/ijms21186906 PMC7555886 32962300

[29] Wegiel J , Gong CX , Hwang YW . The role of DYRK1A in neurodegenerative diseases. FEBS J. 2011;278(2):236‐245. 21156028 10.1111/j.1742-4658.2010.07955.x PMC3052627

[30] Uzdensky AB . Apoptosis regulation in the penumbra after ischemic stroke: expression of pro‐ and antiapoptotic proteins. Apoptosis. 2019;24(9‐10):687‐702. 31256300 10.1007/s10495-019-01556-6

[31] Demyanenko S , Uzdensky A . Profiling of signaling proteins in penumbra after focal photothrombotic infarct in the rat brain cortex. Mol Neurobiol. 2017;54(9):6839‐6856. 27771897 10.1007/s12035-016-0191-x

[32] Yoon HR , Balupuri A , Choi KE , Kang NS . Small molecule inhibitors of DYRK1A identified by computational and experimental approaches. Int J Mol Sci. 2020;21(18):6826. 10.3390/ijms21186826 PMC7554884 32957634

[33] Choi HK , Chung KC . Dyrk1A positively stimulates ASK1‐JNK signaling pathway during apoptotic cell death. Exp Neurobiol. 2011;20(1):35‐44. 22110360 10.5607/en.2011.20.1.35 PMC3213740

[34] Hayakawa R , Hayakawa T , Takeda K , Ichijo H . Therapeutic targets in the ASK1‐dependent stress signaling pathways. P Jpn Acad B‐Phys. 2012;88(8):434‐453. 10.2183/pjab.88.434 PMC3491083 23060232

[35] Guo X , Namekata K , Kimura A , Harada C , Harada T . ASK1 in neurodegeneration. Adv Biol Regul. 2017;66:63‐71. 28882588 10.1016/j.jbior.2017.08.003

[36] Katagiri K , Matsuzawa A , Ichijo H . Regulation of apoptosis signal‐regulating kinase 1 in redox signaling. Methods Enzymol. 2010;474:277‐288. 20609916 10.1016/S0076-6879(10)74016-7

[37] Park MT , Choi JA , Kim MJ , et al. Suppression of extracellular signal‐related kinase and activation of p38 MAPK are two critical events leading to caspase‐8‐ and mitochondria‐mediated cell death in phytosphingosine‐treated human cancer cells. J Biol Chem. 2003;278(50):50624‐50634. 14522966 10.1074/jbc.M309011200

[38] Wang Y , Xia C , Lun Z , Lv Y , Chen W , Li T . Crosstalk between p38 MAPK and caspase‐9 regulates mitochondria‐mediated apoptosis induced by tetra‐alpha‐(4‐carboxyphenoxy) phthalocyanine zinc photodynamic therapy in LoVo cells. Oncol Rep. 2018;39(1):61‐70. 29115534 10.3892/or.2017.6071 PMC5783605

[39] Eriksson M , Pena‐Martinez P , Ramakrishnan R , et al. Agonistic targeting of TLR1/TLR2 induces p38 MAPK‐dependent apoptosis and NFkappaB‐dependent differentiation of AML cells. Blood Adv. 2017;1(23):2046‐2057. 29296851 10.1182/bloodadvances.2017006148 PMC5728277

[40] Li D , Ai Y . Hydrogen saline suppresses neuronal cell apoptosis and inhibits the p38 mitogenactivated protein kinasecaspase3 signaling pathway following cerebral ischemiareperfusion injury. Mol Med Rep. 2017;16(4):5321‐5325. 28849153 10.3892/mmr.2017.7294 PMC5647063

